# On the Number of New World Founders: A Population Genetic Portrait of the Peopling of the Americas

**DOI:** 10.1371/journal.pbio.0030193

**Published:** 2005-05-24

**Authors:** Jody Hey

**Affiliations:** **1**Department of Genetics, Rutgersthe State University of New Jersey, Piscataway, New JerseyUnited States of America; Cornell UniversityUnited States of America

## Abstract

The founding of New World populations by Asian peoples is the focus of considerable archaeological and genetic research, and there persist important questions on when and how these events occurred. Genetic data offer great potential for the study of human population history, but there are significant challenges in discerning distinct demographic processes. A new method for the study of diverging populations was applied to questions on the founding and history of Amerind-speaking Native American populations. The model permits estimation of founding population sizes, changes in population size, time of population formation, and gene flow. Analyses of data from nine loci are consistent with the general portrait that has emerged from archaeological and other kinds of evidence. The estimated effective size of the founding population for the New World is fewer than 80 individuals, approximately 1% of the effective size of the estimated ancestral Asian population. By adding a splitting parameter to population divergence models it becomes possible to develop detailed portraits of human demographic history. Analyses of Asian and New World data support a model of a recent founding of the New World by a population of quite small effective size.

## Abstract 2

A new population-genetic method for assessing human demographic history reveals that the effective size of the founding population of the New World comprised less than 80 individuals.

## Introduction

Archeological evidence, as well as anatomical, linguistic, and genetic evidence, have shown that the original human inhabitants of the Western Hemisphere arrived from Asia during the Late Pleistocene [1–4]. However, there persists uncertainty on the source, within Asia, of peoples who migrated to the New World [5], on the timing of the earliest migration [6–10], and on whether there have been multiple migrations [3,11–13].

For complex historical subjects such as the colonization of the Americas, there are many ways that models can be constructed, examined, and compared. One approach is to develop a portrait based on a particular kind of data, such as linguistic [6], skeletal [14], or archaeological [15] data, or on DNA sequence data from a particular portion of the human genome such as the mitochondria [4,16–19] or the Y chromosome [9]. Yet each source of data has unique sources of variation. In the case of genetic data there occurs a large stochastic variance of the coalescent history among genes that causes different loci to vary widely in levels of genetic variation and in apparent patterns of relationships among populations [20–22]. This stochastic variance is sometimes overlooked, for example in discussions of the histories of the individual DNA sequence haplotypes [18], and it is easy to underestimate the many possible histories that are consistent with a finding that haplotypes are shared by different populations [23–25].

To accommodate the stochastic variance among loci, population geneticists have turned in recent years to Bayesian and likelihood methods that explicitly take into account the range of possible gene tree histories that are consistent with a given dataset [26–30]. For questions on population divergence, the focus has been on models of population splitting in which an ancestral population divides into two descendant populations, after which there may be gene flow between the descendant populations. These “isolation with migration” (IM) models can have a large number of parameters, and they offer the possibility of capturing many of the dynamics that occur in the early stages of population divergence or speciation [30–33].

**Figure 1:**
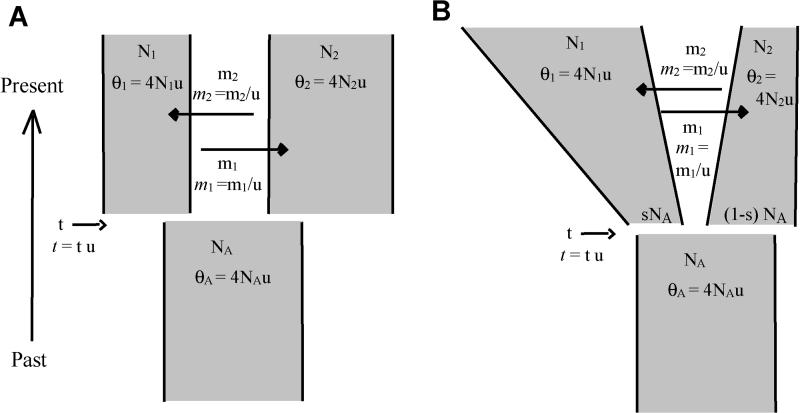
Isolation with Migration Models (A) The basic IM model. The demographic terms are effective population sizes (N_1_, N_2_, and N_A_), gene flow rates (m_1_ and m_2_), and population splitting time (t). Also shown are parameters scaled by the neutral mutation rate (u)*,* as they are actually used in the model fitting. Terms for basic demographic parameters, including N, m, t, and u, are not italicized. Note that the migration parameters are identified by the source of migrants as time goes backward in the coalescent. In other words, the migration rate from population 1 to population 2 (i.e., m_1_) actually corresponds to the movement of genes from population 2 to population 1 as time moves forward. (B) The IM model with changing population size. An additional parameter, *s,* is the fraction of N_A_ that forms N_1_ (i.e., the fraction 1 − *s* gives rise to N_2_)

Figure 1A shows the basic IM model, in which the ancestral and descendant populations each have a constant size. Each of the terms in the model is explained in Table 1. Basic limitations of this model are that it cannot provide details on how descendant populations were founded or whether population sizes have changed. Certainly for human populations there is considerable genetic evidence that population sizes have grown [34–37], and it would be helpful if it were possible to capture information on the sizes of descendant populations as they are formed. For example, if one descendant population formed as a small founder population that later grew to a large size, such dynamics would not be revealed in the fitting of the basic IM model. To allow the study of such histories, an additional parameter has been added to the IM model. Figure 1B shows a model in which an ancestral population splits in two, with the relative sizes of those two new populations reflected in the parameter *s,* where 0 < *s* < 1. At the time of the split, descendant population 1 has size *s*N_A_ from which it moves to size N_1_ at the time of sampling. Similarly, population 2 begins with size (1 − *s*)N_A_ from which it moves to size N_2_ at the time of sampling. Figure 1B depicts one population growing and the other shrinking, but in fact either population is free to either grow or shrink under this model.

**Table 1:**
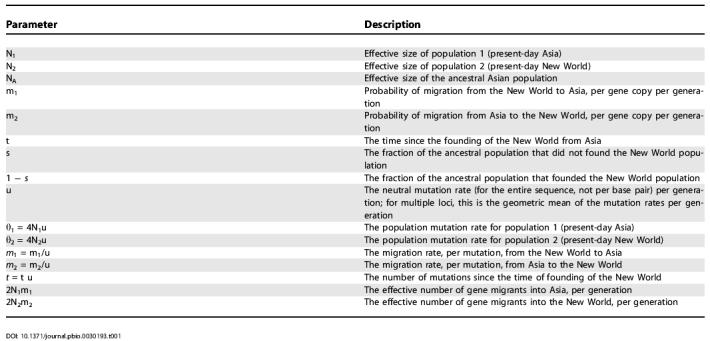
Parameter Summary and Description

**Figure 2:**
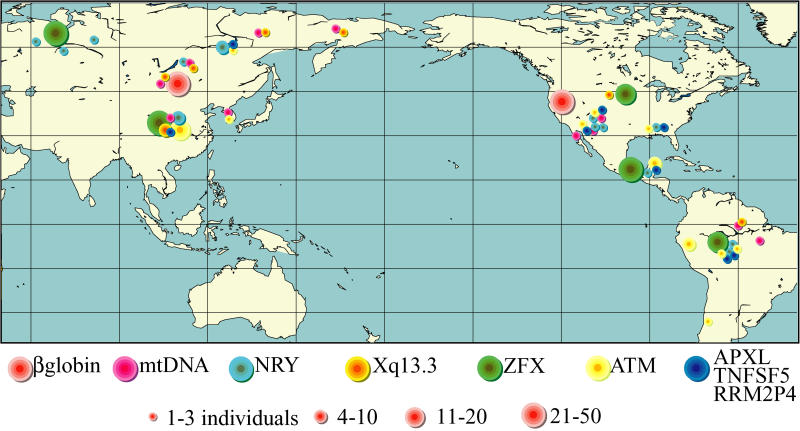
Approximate Geographic Locations, and Sample Sizes per location, for Each Locus Listed in Table 1 In some cases locations are based on actual geographic locations, in other cases the locations are the approximate center of the geographic region occupied by ethnic groups identified in the original references (Table 1).

These models were applied to questions on the founding of New World populations from Asia. A total of nine DNA sequence datasets that included Asian and Native American (Amerind-speaking) samples were drawn from the literature (Figure 2 and Table 1) and analyzed jointly using a procedure that provides posterior probability distributions for each of the model parameters [30,33]. The stochastic variance among loci is clearly evident in the variation of *F*_ST_ values (between Asian and New World samples) observed among the loci. Of the nine loci included in the present study, three have fairly high *F*_ST_ values, while the remainder are either negative (undefined) or near zero (Table 1).

Asian samples were arbitrarily designated as being from population 1 and the New World samples from population 2. In this case, 1 − *s* is the fraction of the ancestral population that founded the New World population. The analyses also require that prior distributions be specified for each model parameter. It was assumed that the New World was founded by a minority of the ancestral Asian population, corresponding to a specified uniform prior distribution for *s* between 0.5 and 1. For the other parameters, flat prior distributions were selected that would span the entire range of the posterior densities (i.e., uninformative priors) [30]. However, in some cases the posterior distributions were quite flat over the highest portions of parameter ranges. In these cases the choice of the upper bound on the prior distribution does affect the posterior distribution, and we are not able to use an uninformative prior distribution. However, parameters can still be estimated on the basis of the locations of peaks in the parameter regions that can be assessed, and the effect of altering the prior distribution on these estimates can be determined.

The overall picture that emerges is one in which the New World was very recently founded by a small number of individuals (effective size of about 70), and then grew by a factor of about 10. The data do suggest that there has been gene exchange between Asia and the New World since that time; however, the likelihood surfaces are quite flat, so confidence in gene flow estimates is low.

## Results

The method assumes that the loci have not been subject to recombination or to directional or balancing selection. For recombination, we used only those loci that showed no evidence of recombination by the four-gamete test [38]. It is possible that this has missed some recombination since the time of common ancestry. Regarding natural selection, the study was limited to loci that had not individually been reported to show evidence of directional or balancing selection. However, it is possible that when considered together, and polymorphism and divergence from chimpanzees are considered under a common neutral model, that there is evidence of selection. An HKA test [39] of the eight loci with estimates of divergence from chimpanzees (Table 2) yielded a *p* value of 0.054, which is nearly statistically significant. This test assumes, as do the models analyzed in this study, that all loci are sampled from the same panmictic population [39], and it is possible that the differing geographic sources of the loci included in the study may have contributed some variation.

**Table 2:**
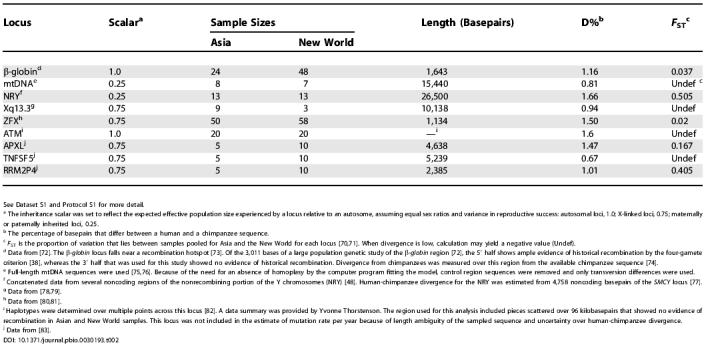
Information on Loci Used in the Study

See Dataset S1 and Protocol S1 for more detail.

^a^ The inheritance scalar was set to reflect the expected effective population size experienced by a locus relative to an autosome, assuming equal sex ratios and variance in reproductive success: autosomal loci, 1.0; X-linked loci, 0.75; maternally or paternally inherited loci, 0.25.

^b^ The percentage of basepairs that differ between a human and a chimpanzee sequence.

^c^ *F*_ST_ is the proportion of variation that lies between samples pooled for Asia and the New World for each locus [70,71]. When divergence is low, calculation may yield a negative value (Undef).

^d^ Data from [72]. The *β-globin* locus falls near a recombination hotspot [73]. Of the 3,011 bases of a large population genetic study of the *β-globin* region [72], the 5′ half shows ample evidence of historical recombination by the four-gamete criterion [38], whereas the 3′ half that was used for this study showed no evidence of historical recombination. Divergence from chimpanzees was measured over this region from the available chimpanzee sequence [74].

^e^ Full-length mtDNA sequences were used [75,76]. Because of the need for an absence of homoplasy by the computer program fitting the model, control region sequences were removed and only transversion differences were used.

^f^ Concatenated data from several noncoding regions of the nonrecombining portion of the Y chromosomes (NRY) [48]. Human-chimpanzee divergence for the NRY was estimated from 4,758 noncoding basepairs of the *SMCY* locus [77].

^g^ Data from [78,79].

^h^ Data from [80,81].

^i^ Haplotypes were determined over multiple points across this locus [82]. A data summary was provided by Yvonne Thorstenson. The region used for this analysis included pieces scattered over 96 kilobasepairs that showed no evidence of recombination in Asian and New World samples. This locus was not included in the estimate of mutation rate per year because of length ambiguity of the sampled sequence and uncertainty over human-chimpanzee divergence.

^j^ Data from [83].

**Figure 3:**
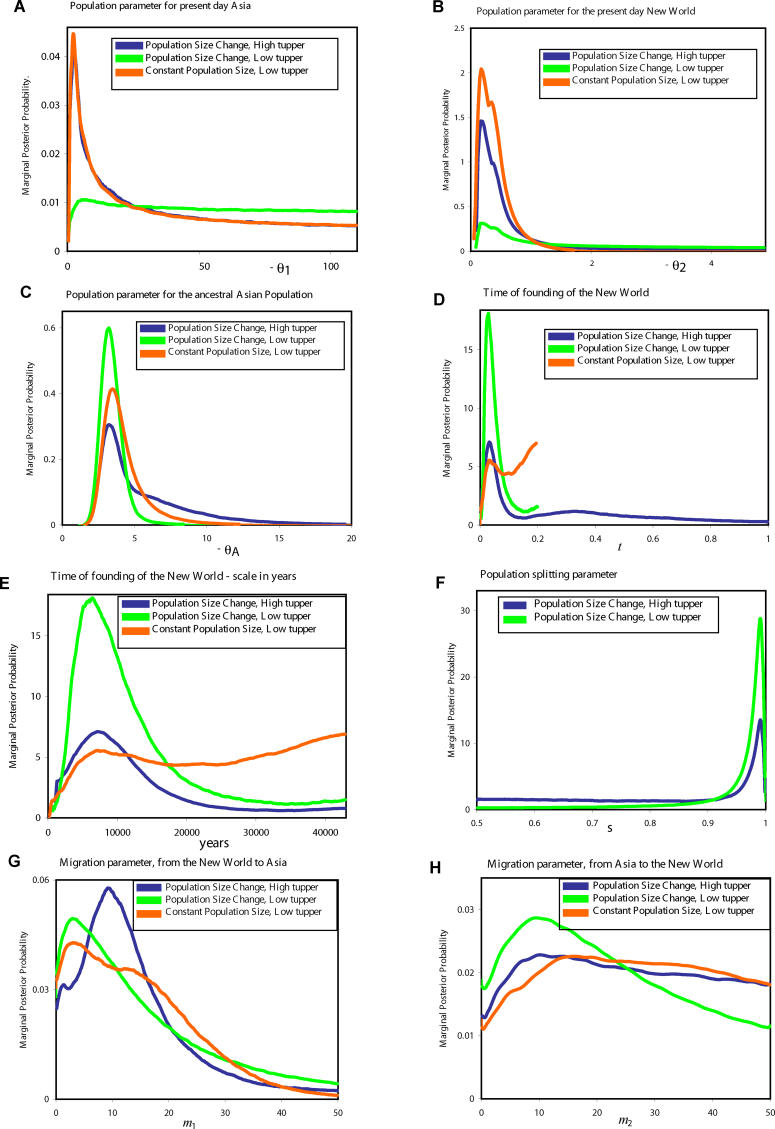
Marginal Posterior Probability Densities Probability densities for each of the parameters described in Figure 1 are shown, as follows: (A) θ_1_; (B) θ_2_; (C) θ_A_; (D) *t* (i.e., t/u); (E) t shown on a scale of years over the range corresponding to a maximum *t* value of 0.2; (F) *s;* (G) *m*_1_; and (H) *m*_2_. The analysis in which a high upper limit on the prior distribution for *t* was used is identified as “high *t_upper_*,” while those analyses with a smaller upper limit on the prior distribution of *t* are identified as “low *t_upper_*.” Each curve is based upon the results of multiple simulations over millions of Markov chain updates (see Materials and Methods), and is plotted over the specified prior range of that parameter.

The estimated posterior distributions are shown in Figure 3. For the initial analysis, allowing for exponential population size changes, the posterior distribution for *t* yielded both a major and a minor peak (the curve for *t* with a high *t*_upper_, Figure 3D). Given the mutation rate estimates (see Table 1), the location of the major peak (*t* = 0.032) corresponds to 7,130 y, whereas the location of the minor peak (*t* = 0.27) corresponds to 44,400 y. Given the remote possibility of such an ancient time as the latter, analyses were also done with a smaller upper bound on *t* of 0.2 (identified as “low *t*_upper_” in Figure 3), which corresponds to 33,000 y. Analyses were done with this reduced upper limit for *t* for both models in Figure 1, allowing for population size change and for the case of fixed population sizes. In the case of constant population sizes, the distribution for *t* shows a peak (*t* = 0.038) very near those for the analyses under population size change; however, the highest posterior density is found at the upper limit of *t*. When the constant population size model was run with a higher upper limit on *t,* the posterior distribution showed the same low value peak as well as a steadily rising curve for higher values of *t* (unpublished data).

The archaeological portrait of early New World populations has largely centered around widespread Clovis sites that have an earliest estimated age of about 13,000 y before the present [15,40,41]. The oldest generally agreed upon New World archaeological date is from the non-Clovis Monte Verde site in Southern Chile, which has been dated to about 14,000 y before the present [10,42,43]. Clearly the time points associated with our estimates of *t* are more recent than expected, given the archaeological estimates. However, these distributions do span the time periods that have been most discussed. For example, a time of 14,000 y has a relatively high probability in each of the analyses (Figure 3E). Given that people have lived in the New World probably for only several hundred generations, it is noteworthy both that the posterior densities for *t* do show clear peaks in the expected time period and that the probability estimates drop to zero as *t* approaches zero. In other words, the data contain a clear signal of a nonzero, albeit recent, founding time of New World populations.

With regard to migration, each of the three analyses show nonzero peaks for both directions of gene flow. These may well reflect the occurrence of more than one episode of migration to the New World. For example, it has been suggested on the basis of mitochondrial DNA haplotypes and glaciation history that an initial migration along a coastal route may have been followed later by another migration, possibly through an ice-free noncoastal corridor [13]. However, the posterior distributions shown here have little resolution, as all of the curves for *m*_1_ and *m*_2_ are broad, and all have high probability at the lower limit of resolution, indicating that zero gene flow is nearly as well supported by the data as are nonzero gene flow levels.

The ancestral population parameter, θ_A_, shows a relatively narrow distribution with a very consistent peak location across the three analyses. These attributes are partly to be expected, given that the very large majority of the variation in the samples is older than *t*. In effect, more information is available for θ_A_ than for the other parameters. The estimated effective size of the ancestral population is about 9,000 (Table 3), which is roughly consistent with previous estimates for Asian samples [44]. The current Asian population parameter (θ_1_) revealed broad distributions and estimates that are near those for the ancestral population. Although the estimates of current effective size in Asia vary among the analyses (Table 3), they are all fairly close to the ancestral size estimates, suggesting that there has not been much population growth in Asia since *t*. Also consistent with the apparent constancy of population size is the distribution of *s,* the splitting parameter, which shows a peak at 0.992, signifying that only a small portion (less than 1%) of the ancestral Asian population left to found the New World population.

**Table 3:**
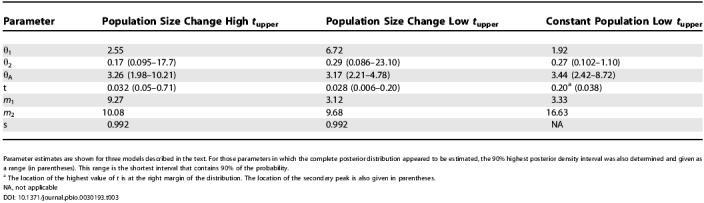
Model Parameter Estimates

Parameter estimates are shown for three models described in the text. For those parameters in which the complete posterior distribution appeared to be estimated, the 90% highest posterior density interval was also determined and given as a range (in parentheses). This range is the shortest interval that contains 90% of the probability.

^a^ The location of the highest value of *t* is at the right margin of the distribution. The location of the secondary peak is also given in parentheses.

NA, not applicable

In contrast to the Asian population, the New World population parameter (θ_2_) is much smaller, and suggests a recent New World effective population size of less than 1,000 (Table 3). However, given the estimate of the effective size of the founding New World population (about 70; Table 4), the overall picture is of a nearly 10-fold growth in the New World effective size since *t*.

**Table 4:**
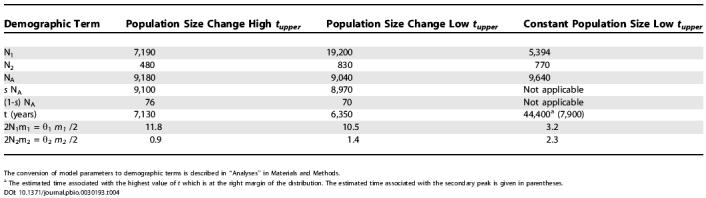
Estimates of Demographic Quantities

The conversion of model parameters to demographic terms is described in “Analyses” in Materials and Methods.

^a^ The estimated time associated with the highest value of *t* which is at the right margin of the distribution. The estimated time associated with the secondary peak is given in parentheses.

In order to gain a sense of how consistent the data actually are with the model and the parameter estimates, 500 simulated datasets were generated under the model in Figure 1B, with sample sizes and true parameter values (see Table 2, column 3) that were the same as for the actual data. From each simulated dataset, the average number of pairwise differences between sequences were calculated within each population (Asia and the New World) and between these populations. The average of these values from the 500 simulated datasets, and the observed values from the actual data, are shown in Table 5. In general, the observed and expected values are similar; however, one consistent pattern of departure is that the data from the New World, for most loci, show more variation by this measure than were found in the simulated data.

**Table 5:**
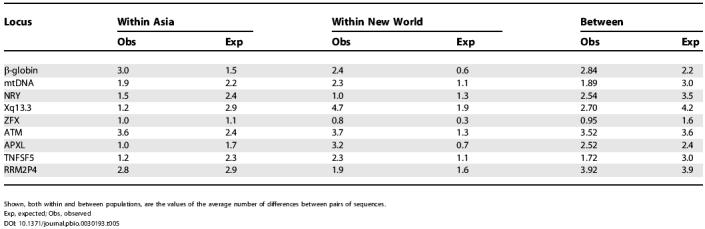
Contrasting Observed and Expected Levels of Variation

Shown, both within and between populations, are the values of the average number of differences between pairs of sequences.

Exp, expected; Obs, observed

## Discussion

The method described is one of several new approaches that can glean information about ancestral population sizes [30,45–47]. By including a new parameter for population splitting, it is possible to generate estimates not only of the size of the ancestral population, but also of the founding size of each founder population.

Taken together, the analyses in this study suggest a recent founding of the New World Amerind-speaking peoples by a small population of effective size near 70, followed by population growth in the New World. It is interesting that the analyses do not suggest much population size change in Asia since the time of the founding of the New World population. Given the very broad distributions for θ_1_, it is possible that the true value of this parameter is much higher than suggested by the peak location, and that there has been considerable population growth in Asia. The analyses reveal very broad distributions for migration parameters, and although the peak locations suggest that gene flow has been fairly high (2Nm values greater than 1; see Table 3), the estimated probabilities of migration rates having been zero are also high (Figure 3G and 3H). Also, because Eskimo-Aleut and Na Déne speakers were not included in this study, the question of separate migrations for these groups has not been addressed [3].

As parameter-rich as the method is, neither this nor any mathematical model can be expected to fully represent the complex history of two related populations. However, the same is essentially true of narrative models, as investigators are always constrained by limited data and the need to keep explanations as simple as possible given their data. In this light, the IM model provides a fairly complete framework for some oft-debated questions on human history. With the addition of a new parameter, the IM framework can now also be used to address questions about the founding size of populations and of population size change.

In the context of human demographic history, the most problematic assumption under the IM model is that each population is panmictic. Certainly this is not the case today, and it is likely to have even been less true in times past. This raises the general and important question of how local patterns of population structure affect regional or global estimates of diversity [44,48,49]. Although this question cannot be answered here, the analyses do suggest that some kinds of departures from panmixia have not occurred. For example, if the New World had been founded by a local population that had long been separated from other Asian populations, then the estimate of *t* would be expected to reflect this older population structure, rather than the founding of the New World. Our generally low estimates of *t* argue against this scenario. Similarly, if the sampled Asian populations had been highly structured, with many long-separated local populations, then this would have inflated the estimates of N_A_ and N_1_, respectively. However, the generally low estimates of effective population size argue against this particular kind of population structure.

The analyses presented here share with some other genetic studies estimated dates for the peopling of the Americas that are more recent than archeologically based estimates [8,9,16]. However, the difficulty of estimating such recent events using genetic data alone should not be overestimated [18]. When considering human populations within the past few tens of thousands of years, two gene copies that share the same haplotype will often have had a common ancestor far longer ago than any of the dates in question. Similarly, genetic evidence on the peopling of the Americas has been interpreted both as consistent with multiple migrations [12] and as indicating just a single founder event [16,19,50]. Divergent interpretations are understandable, given that a finding of two populations that share sequence haplotypes at a locus can be taken as evidence of two quite different models: (1) a recent population separation; or (2) gene exchange between populations.

The available data do not yet allow precise estimates of founding time nor of whether there has been gene flow between the New World and Asia following the initial founding event. However, the new method implements a parameter-rich model of divergence and has the potential to recover the history of complex divergence processes. The method can also be applied to a large number of loci, with large sample sizes, and in the future can be expected to provide increasingly detailed portraits of human population divergence.

## Materials and Methods

### 

#### Selected loci and samples

Given the prevailing model of the founding of New World populations via a Bering land bridge, the descendant populations were defined as the Amerind speakers of the New World and the peoples of northeastern Asia. Greenberg et al. [3] proposed that New World populations include three linguistic groups (Eskimo-Aleut, Na Déne, and Amerind), each associated with a separate episode or period of migration. Because of the limited number of published comparative DNA sequence studies that include samples from Eskimo-Aleut and Na Déne group, the present study was limited to samples from Amerind-speaking populations. Asian samples were limited to those from China, Mongolia, Korea, and Siberia. These are partly arbitrary boundaries selected as a balance between the need to include as many loci as possible and uncertainty about the present locations of descendants of those Asian populations that gave rise to the founders of the New World.

The model fitting requires data from loci that do not show evidence of recombination and that do not show clear evidence of directional or balancing selection. All available datasets from the literature that met these criteria and that had multiple DNA sequences from both of the designated sample regions were selected. The selected loci are listed in Table 1. The input data file is provided in Dataset S1, and a list of sample locations is provided in Protocol S1.

#### Model development.

At the center of the method for estimating the parameters is an expression for the posterior probability distribution of model parameters Θ, given the data. For the case of multiple loci



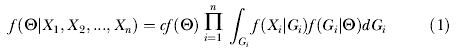



where Θ refers to the vector of parameters of the model, *X_i_* refers to the data for locus *i,* and *G*_i_ is the genealogy for locus *i* [33]. With *n* loci, the full set of parameters includes six or seven demographic parameters, depending on the inclusion of *s,* as well as *n* locus-specific mutation rate scalars [33]. A genealogy includes the topology of an ultrametric tree, the associated coalescence times, and the times of migrations on each branch of the tree [30]. For a given locus *i,* the probability *f*(*X*_i_|*G*_i_) is calculated using the mutation model for that locus and the branch lengths in the genealogy. The probability *f*(*G*_i_|Θ) is calculated using expressions from basic coalescent theory [30,51–55]. By integrating over all possible genealogies that are consistent with the data, the results obtained are not conditioned on any particular estimate of the genealogy, and they necessarily incorporate all of the stochastic variance that arises among independent loci under the model.

The integration in Equation 1 cannot be solved directly for any but the simplest of models, but it can be approximated using a Markov chain simulation [56]. This approach was originally applied to the IM model by Nielsen and Wakeley [30], and then augmented to include multiple loci [33] and additional mutation models [32,57].

Over the course of a simulation the genealogy for a given locus varies for topology, branch lengths, and migration times. However, the probability of the data for a locus given a particular genealogy, *f*(*X*_i_|*G*_i_), depends only upon the branch lengths and the mutation model for that locus [30]. Although inclusion of *s* will affect the genealogies that arise in the course of the simulation, there will be no effect on the calculation of the probability of the data for a given genealogy (i.e., *f*(*X*_i_|*G*_i_) is not a function of *s*), and thus including *s* has no effect on the applicability of the method to diverse mutation models. In contrast, the probability of a genealogy given a set of parameter values, *f*(*G*_i_|Θ), depends strongly on *s* because the probability of individual coalescent and migration times are functions of population size.

The calculation of *f*(*G*_i_|Θ) is most directly done by taking the product of the probabilities of each of the coalescent and migration events that occur in the genealogy. Griffiths and Tavare [55] developed the general theory for the probability distribution of coalescence times when the population size is changing. Given a function *v*(τ)= N_τ_/N_0_ of the population size at time τ, relative to that at time 0, they provide a general expression for the distribution of coalescent times. For population 1, the effective size goes from N_1_ at time zero, to *s*N_A_ at time t. If it is assumed that the size change is exponential over this period, then for population 1,



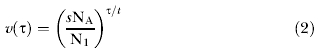



and for population 2,



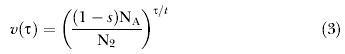



One additional complication that arises is that when the population is growing exponentially back into the past (decreasing in size as time moves forward), there is a finite probability that the time to coalescence will be infinity [58]. Thus, for population 1 when *s*N_A_ is less than N_1_, it is necessary to calculate the probability of coalescence time conditioned on there being a coalescent event.

Migration under an exponentially changing population size can also be incorporated under this same framework with two changes. First, unlike coalescence, where the rate is inversely proportional to population size, the rate of migration is directly proportional to population size. Second, as time goes backward in the coalescent, the migration rate from population 1 to population 2 (i.e., *m*_1_) actually corresponds to the movement of genes from population 2 to population 1 as time moves forward. This means that in the coalescent under changing population size, we expect that the migration rate from population 1 to 2 will vary with the size of population 2. Thus the corresponding relative rate function for migration from population 2 to population 1 is



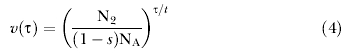



and for migration in the reverse direction it is



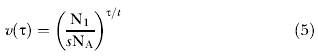



These intensity functions for coalescence and migration were used to develop an expression for *f*(*G*_i_|Θ) that includes *s,* and that could be directly incorporated into the update criteria for all of the demographic, mutation, and inheritance scalars described in Hey and Nielsen [33]. Also needed, in order to allow for changing population size, are the update criteria for *s* and the update criteria for the genealogies. For *s,* updates are drawn from a uniform distribution over the user-specified prior range (e.g., in the current study, an interval within the range of 0.5 to 1). An update from *s* to s* will affect the probability of all genealogies and thus has an acceptance probability, under the Metropolis Hastings criterion, of



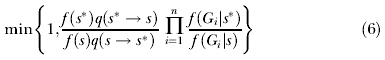



where *n* is the number of loci and *G*_i_ is the current genealogy for locus *i* (see Equation 3 in Hey and Nielsen [33]). If we assume a uniform prior distribution for *s,* such that the prior probability of *s, f(s),* is constant for all *s,* and if we choose updates such that the *q*(s* → *s*) = *q*(*s* → s*) [30], then this simplifies to



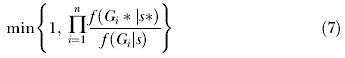



For genealogy updates the same proposal distribution of genealogies that was used in the case without *s* was retained, and then this proposal distribution was incorporated into the update criteria [59]. If *f*(*G*_i_|*s*) denotes the probability of the genealogy for locus *i,* given the other parameters including *s,* and *f*(*G*_i_) is the Hastings term for the proposal probability of the genealogy for locus *i,* given the other parameters1 excluding *s,* then the update criteria for the genealogy for locus *i* is



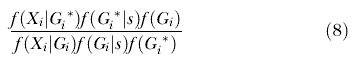



#### Performance.

**Figure 4:**
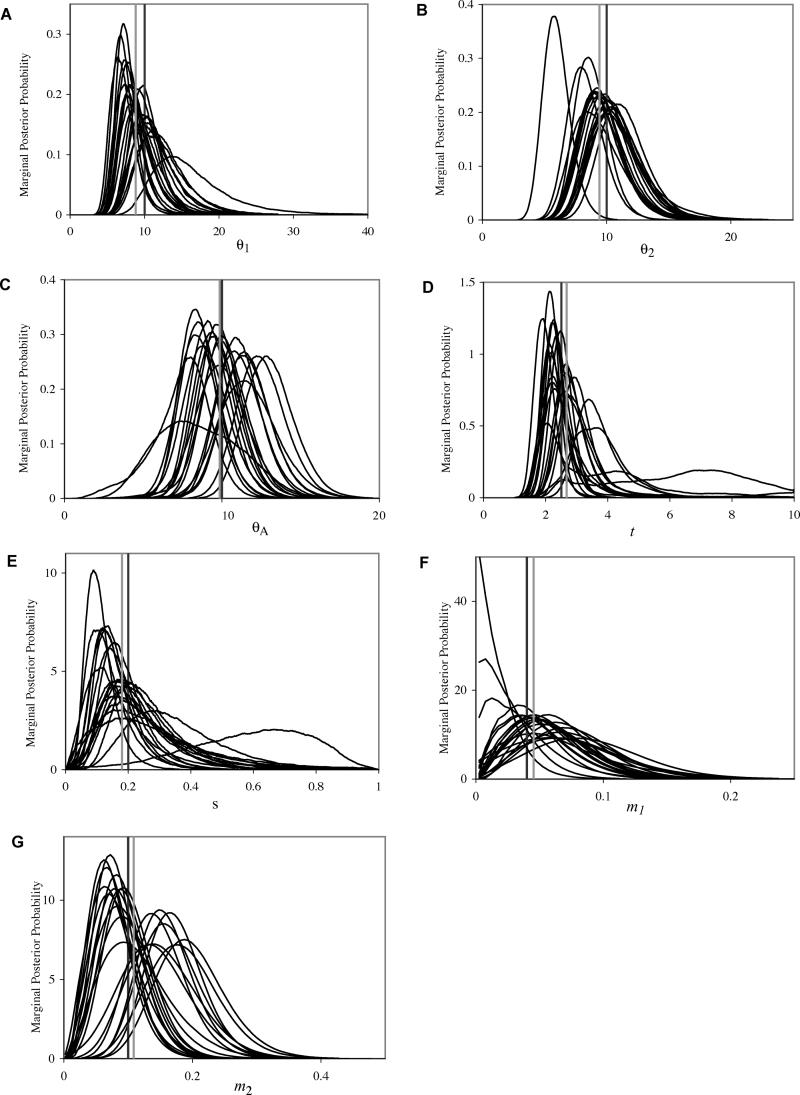
The Marginal Densities Obtained by Fitting the Model with Population Size Change to Simulated Data The input parameters for the simulations were as follows: (A) θ_1_ = 10; (B) θ_2_ = 10; (C) θ_A_ = 10; (D) *t* =2.5, (E) *s* = 0.2, (F) *m*_1_= 0.04; (G) *m*_2_= 0.2 ; and *t* = 5 (t/2N_A_ = 0.5). For each simulated dataset, coalescent simulations were done for each of 20 loci with identical mutation rates under an infinite sites mutation model, each with sample sizes of 10 for each of the two populations. Each simulated dataset was analyzed using wide uniform prior distributions for each parameter. Each analysis began with a burn-in period of 300,000 steps followed by a primary chain of 3 million to 10 million steps. The curves for parameters θ_1_ through *m_2_* are shown in (A) through (G), respectively. For each figure, the true parameter value used in the simulations is shown as a black vertical bar, and the mean of the estimates for the 20 simulations (based on peak locations) is shown as a gray vertical bar.



The IM computer program [33] was modified to include the additional parameter. The program is available from http://lifesci.rutgers.edu/~heylab/HeylabSoftware.htm#IM. For the Markov chain simulation that is implemented by the program, it is difficult to assess how well the method works, because of the need to generate large numbers of simulated datasets and because of the long run times required [33]. To conduct testing, a program was written to generate simulated datasets under the models in Figure 1. Datasets were simulated in groups of 10 or 20, each having 10–20 loci, for a given set of parameter values, and for a range of parameter values. Figure 4 shows the marginal posterior densities estimated from each of 20 independent simulations for a case of modest population growth with the following parameter values. θ_1_ = 10; θ_2_, = 10; θ_A_ = 10; *t* = 2.5; *s* = 0.2; *m*_1_ = 0.04; and *m*_2_ = 0.1. For each parameter, the mean of the 20 estimates is shown, and in general these are fairly close to the true value, though there is considerable variance for the peak locations in individual runs. To test whether the locations of these distributions are consistent with the true values of the parameters (i.e., the values used in the simulations), probabilities were combined by treating each simulation as an independent test of the same hypothesis [60]. For each posterior density *p_i_, i* = 1,…,20, is the chance that a parameter value is more extreme (i.e., departs more from the mean of the distribution) than is the actual true value. That is, if *x* is the area of the curve to the left of the true value then *p_i_* = 2*x* if *x* < 0.5 and *p_i_* = 2(1 − *x*) if *x* > 0.5. If the *p_i_*'s are uniformly distributed, then the quantity

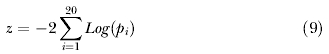



is χ^2^ distributed with 40 degrees of freedom (i.e., two times the number of densities). The *z* values were as follows: θ_1_, 35.5; θ_2_, 26.4; θ_A_, 41.7; *t,* 41.1; *s*, 26.4; *m*_1_, 29.9; *m*_2_, 44.1; and the mean of the seven values was 35.0. In the corresponding χ^2^ distribution, 90% of the probability mass falls above 29.05; 50% falls above 39.3; and 10% falls above 51.8 [61]. Clearly these values are not entirely independent of each other, but they all fall in the middle of the χ^2^ distribution with a mean (35.0) close to the 50% point of the χ^2^ distribution (39.3).

From these simulations, and many others (additional results provided in Protocol S1), it is clear that sample sizes do need to be large for the posterior distributions to be informative. With data from fewer than five loci or fewer than ten individuals per population per locus, it is often the case that distributions are very flat or that there are multiple peaks. There is a tradeoff in sampling effort required for different kinds of histories. When *t* is small, sampling effort should be shifted to larger sample sizes per locus, whereas when *t* is large, sampling effort should be shifted toward more loci. This tradeoff is a byproduct of the fact that the stochastic variance among loci, that is associated with coalescent and migration events in genealogies at times near *t,* goes up as *t* increases. Another tradeoff that arises is between *s* and the migration rate parameters. Just as the frequency of polymorphic sites can be used to estimate changes in population size [62], it can also be appreciated that the information for *s* must reside in the distribution of times of node intervals in the descendant populations. Migration can have dramatic effects on node interval times within populations. In practice, via simulation, the method does not resolve a sharp peak for *s* for populations that have had more than moderate amounts of migration (e.g., 2Nm values are greater than 0.5; see Protocol S1).

#### Analyses

Each of the three analyses were done using at least three independent runs, with ten or more independent chains under Metropolis coupling [33] as described by Geyer [63]. Each chain was initiated with a burn-in period of 100,000 updates, and the total run length of each analysis was between 10 million and 30 million updates. The mixing properties of individual runs were monitored by measuring the autocorrelation of individual parameters over the course of the run, and by estimating the effective sample size for each of the parameters as a function of the autocorrelation estimates (see p. 499 in [64]). Analyses were taken to have converged upon the stationary distribution if independent runs generated similar distributions, with each having a lowest effective sample size of 50 for the time parameter (the parameter to show the slowest rate of mixing).

To convert estimates of parameters that include the mutation rate to more easily interpreted units, a value of 6 million y since the splitting of human and chimpanzee lineages was used [65–69]. The geometric mean of the human-chimpanzee DNA sequence divergence of each locus, except *ATM* (see Table 2), was calculated and then used as a molecular clock calibration for converting the estimate of the time parameter, *t*, to divergence in years. The geometric mean mutation rate across these loci was estimated to be 4.66 × 10^−6^ mutations per year. The geometric mean is used rather than an arithmetic mean, because under the multilocus model, the mutation rate by which demographic parameters are scaled is the geometric mean of the individual locus-specific mutation rates [33].

To convert the estimates of the population mutation rate parameters (θ_1_, θ_2_, and θ_A_) to estimates of effective population size (N_1_, N_2_, and N_A_, respectively) a measure of mutation rate on a scale of generations is needed. Thus, an assumption was made of 20 y per generation, and the geometric mean divergence between humans and chimpanzees for each species contrast was divided by 12 million y then multiplied by 20 y per generation. These calculations yielded a geometric mean value of 9.32 × 10^−5^ mutations per generation. These mutation rate values were then used to convert individual θ estimates to effective population size estimates (i.e., θ = 4Nu, and N = θ/4u).

Migration parameters in the model can be used to obtain population migration rate estimates (i.e., *M* = 2Nm*,* the product of the effective number of gene copies and the per gene copy migration rate) using an estimate of the population mutation rate (θ = 4Nu). Thus θ × *m*/2 = (4Nu × m/u)/2 = 2Nm [32].

## Supporting Information

Dataset S1 Peopling of Americas Data File: Nine Loci This is the input file that contains all of the data and that was analyzed using the IM computer program.
(582 KB TXT).

Protocol S1 Additional Simulations and List of Sample Locations (92 KB DOC).

## Abbreviation

IM: isolation with migration

## References

[pbio-0030193-b01] Brace CL, Nelson AR, Seguchi N, Oe H, Sering L (2001). Old World sources of the first New World human inhabitants: A comparative craniofacial view. Proc Natl Acad Sci U S A.

[pbio-0030193-b02] Goebel T (1999). Pleistocene human colonization of Siberia and peopling of the Americas: An ecological approach. Evol Anthropol.

[pbio-0030193-b03] Greenberg JH, Turner CG, Zegura SL (1986). The settlement of the Americas: A comparison of the linguistic, dental and genetic evidence. Curr Anthropol.

[pbio-0030193-b04] Wallace DC, Garrison K, Knowler WC (1985). Dramatic founder effects in Amerindian mitochondrial DNAs. Am J Phys Anthropol.

[pbio-0030193-b05] Goebel T, Waters MR, Dikova M (2003). The archaeology of Ushki Lake, Kamchatka, and the Pleistocene peopling of the Americas. Science.

[pbio-0030193-b06] Nichols J (1990). Linguistic diversity and the first settlement of the New World. Language.

[pbio-0030193-b07] Nettle D (1999). Linguistic diversity of the Americas can be reconciled with a recent colonization. Proc Natl Acad Sci U S A.

[pbio-0030193-b08] Starikovskaya YB, Sukernik RI, Schurr TG, Kogelnik AM, Wallace DC (1998). mtDNA diversity in Chukchi and Siberian Eskimos: Implications for the genetic history of Ancient Beringia and the peopling of the New World. Am J Hum Genet.

[pbio-0030193-b09] Lell JT, Brown MD, Schurr TG, Sukernik RI, Starikovskaya YB (1997). Y chromosome polymorphisms in native American and Siberian populations: Identification of native American Y chromosome haplotypes. Hum Genet.

[pbio-0030193-b10] Fiedel SJ (2000). The peopling of the New World: Present evidence, new theories, and future directions. J Archaeol Res.

[pbio-0030193-b11] Tarazona-Santos E, Santos FR (2002). The peopling of the americas: A second major migration?. Am J Hum Genet.

[pbio-0030193-b12] Lell JT, Sukernik RI, Starikovskaya YB, Su B, Jin L (2002). The dual origin and Siberian affinities of Native American Y chromosomes. Am J Hum Genet.

[pbio-0030193-b13] Schurr TG, Sherry ST (2004). Mitochondrial DNA and Y chromosome diversity and the peopling of the Americas: Evolutionary and demographic evidence. Am J Human Biol.

[pbio-0030193-b14] Gonzalez-Jose R, Dahinten SL, Luis MA, Hernandez M, Pucciarelli HM (2001). Craniometric variation and the settlement of the Americas: Testing hypotheses by means of R-matrix and matrix correlation analyses. Am J Phys Anthropol.

[pbio-0030193-b15] Taylor RE, Haynes CV, Stuiver M (1996). Clovis and Folsom age estimates: Stratigraphic context and radiocarbon calibration. Antiquity.

[pbio-0030193-b16] Bonatto SL, Salzano FM (1997). Diversity and age of the four major mtDNA haplogroups, and their implications for the peopling of the New World. Am J Hum Genet.

[pbio-0030193-b17] Merriwether DA, Clark AG, Ballinger SW, Schurr TG, Soodyall H (1991). The structure of human mitochondrial DNA variation. J Mol Evol.

[pbio-0030193-b18] Malhi RS, Eshleman JA, Greenberg JA, Weiss DA, Schultz Shook BA (2002). The structure of diversity within New World mitochondrial DNA haplogroups: Implications for the prehistory of North America. Am J Hum Genet.

[pbio-0030193-b19] Silva WA, Bonatto SL, Holanda AJ, Ribeiro-Dos-Santos AK, Paixao BM (2002). Mitochondrial genome diversity of Native Americans supports a single early entry of founder populations into America. Am J Hum Genet.

[pbio-0030193-b20] Takahata N, Nei M (1985). Gene genealogy and variance of interpopulational nucleotide differences. Genetics.

[pbio-0030193-b21] Hudson RR, Futuyma D, Antonovics J (1990). Gene genealogies and the coalescent process. Oxford Surveys in Evolutionary Biology.

[pbio-0030193-b22] Hudson RR, Turelli M (2003). Stochasticity overrules the “three-times rule”: Genetic drift, genetic draft, and coalescence times for nuclear loci versus mitochondrial DNA. Evolution.

[pbio-0030193-b23] Knowles LL, Maddison WP (2002). Statistical phylogeography. Mol Ecol.

[pbio-0030193-b24] Templeton AR (2004). Statistical phylogeography: Methods of evaluating and minimizing inference errors. Mol Ecol.

[pbio-0030193-b25] Hey J, Machado CA (2003). The study of structured populations—New hope for a difficult and divided science. Nat Rev Genet.

[pbio-0030193-b26] Felsenstein J (1992). Estimating effective population size from samples of sequences: A bootstrap Monte Carlo integration method. Genet Res.

[pbio-0030193-b27] Kuhner MK, Yamato J, Felsenstein J (1995). Estimating effective population size and mutation rate from sequence data using Metropolis-Hastings sampling. Genetics.

[pbio-0030193-b28] Griffiths RC, Tavare S (1994). Ancestral inference in population genetics. Stat Sci.

[pbio-0030193-b29] Beerli P, Felsenstein J (1999). Maximum-likelihood estimation of migration rates and effective population numbers in two populations using a coalescent approach. Genetics.

[pbio-0030193-b30] Nielsen R, Wakeley J (2001). Distinguishing migration from isolation. A Markov chain Monte Carlo approach. Genetics.

[pbio-0030193-b31] Wakeley J, Hey J, DeSalle R, Schierwater B (1998). Testing speciation models with DNA sequence data. Molecular Approaches to Ecology and Evolution.

[pbio-0030193-b32] Hey J, Won Y-J, Sivasundar A, Nielsen R, Markert JA (2004). Using nuclear haplotypes with microsatellites to study gene flow between recently separated Cichlid species. Mol Ecol.

[pbio-0030193-b33] Hey J, Nielsen R (2004). Multilocus methods for estimating population sizes, migration rates and divergence time, with applications to the divergence of Drosophila pseudoobscura and D. persimilis. Genetics.

[pbio-0030193-b34] Pritchard JK, Seielstad MT, Perez-Lezaun A, Feldman MW (1999). Population growth of human Y chromosomes: A study of Y chromosome microsatellites. Mol Biol Evol.

[pbio-0030193-b35] Harpending HC, Batzer MA, Gurven M, Jorde LB, Rogers AR (1998). Genetic traces of ancient demography. Proc Natl Acad Sci U S A.

[pbio-0030193-b36] Sherry ST, Rogers AR, Harpending H, Soodyall H, Jenkins T (1994). Mismatch distributions of mtDNA reveal recent human population expansions. Hum Biol.

[pbio-0030193-b37] Watkins WS, Ricker CE, Bamshad MJ, Carroll ML, Nguyen SV (2001). Patterns of ancestral human diversity: An analysis of alu-insertion and restriction-site polymorphisms. Am J Hum Genet.

[pbio-0030193-b38] Hudson RR, Kaplan NL (1985). Statistical properties of the number of recombination events in the history of a sample of DNA sequences. Genetics.

[pbio-0030193-b39] Hudson RR, Kreitman M, Aguadé M (1987). A test of neutral molecular evolution based on nucleotide data. Genetics.

[pbio-0030193-b40] Meltzer DJ (1995). Clocking the first Americans. Annu Rev Anthropol.

[pbio-0030193-b41] Anderson DG, Faught MK (2000). Palaeoindian artefact distributions: Evidence and implications. Antiquity.

[pbio-0030193-b42] Dillehay TD, editor (1996). Monte Verde: A late Pleistocene settlement in Chile. Vol 2: The archaeological context and interpretation.

[pbio-0030193-b43] Meltzer DJ (1997). Monte Verde and the Pleistocene peopling of the Americas. Science.

[pbio-0030193-b44] Tishkoff SA, Verrelli BC (2003). Patterns of human genetic diversity: Implications for human evolutionary history and disease. Annu Rev Genomics Hum Genet.

[pbio-0030193-b45] Rannala B, Yang Z (2003). Bayes estimation of species divergence times and ancestral population sizes using DNA sequences from multiple loci. Genetics.

[pbio-0030193-b46] Rosenberg NA, Feldman MW, Slatkin M, Veuille M (2002). The relationship between coalescence times and population divergence times. Modern developments in theoretical population genetics.

[pbio-0030193-b47] Takahata N, Lee SH, Satta Y (2001). Testing multiregionality of modern human origins. Mol Biol Evol.

[pbio-0030193-b48] Hammer MF, Blackmer F, Garrigan D, Nachman MW, Wilder JA (2003). Human population structure and its effects on sampling y chromosome sequence variation. Genetics.

[pbio-0030193-b49] Zietkiewicz E, Yotova V, Gehl D, Wambach T, Arrieta I (2003). Haplotypes in the dystrophin DNA segment point to a mosaic origin of modern human diversity. Am J Hum Genet.

[pbio-0030193-b50] Merriwether DA, Rothhammer F, Ferrell RE (1995). Distribution of the four founding lineage haplotypes in Native Americans suggests a single wave of migration for the New World. Am J Phys Anthropol.

[pbio-0030193-b51] Kingman JFC (1982). The coalescent. Stochastic Processes Appl.

[pbio-0030193-b52] Kingman JFC (1982). On the genealogy of large populations. J Appl Probab 19A.

[pbio-0030193-b53] Tavare S (1984). Line-of-Descent and genealogical processes, and their applications in population genetics models. Theor Popul Biol.

[pbio-0030193-b54] Hudson RR (1983). Properties of a neutral allele model with intragenic recombination. Theor Popul Biol.

[pbio-0030193-b55] Griffiths RC, Tavare S (1994). Sampling theory for neutral alleles in a varying environment. Philos Trans R Soc Lond B Biol Sci.

[pbio-0030193-b56] Gilks WR, Richardson S, Spiegelhalter DJ (1996). Markov chain Monte Carlo in practice.

[pbio-0030193-b57] Palsbøll PJ, Berube M, Aguilar A, Notarbartolo-Di-Sciara G, Nielsen R (2004). Discerning between recurrent gene flow and recent divergence under a finite-site mutation model applied to North Atlantic and Mediterranean Sea fin whale *(Balaenoptera physalus)* populations. Evolution Int J Org Evolution.

[pbio-0030193-b58] Kuhner MK, Yamato J, Felsenstein J (1998). Maximum likelihood estimation of population growth rates based on the coalescent. Genetics.

[pbio-0030193-b59] Hastings WK (1970). Monte Carlo sampling methods using Markov chains and their applications. Biometrika.

[pbio-0030193-b60] Fisher RA (1954). Statistical methods for research workers.

[pbio-0030193-b61] Rohlf FJ, Sokal RR (1981). Statistical tables.

[pbio-0030193-b62] Tajima F (1989). The effect of change in population size on DNA polymorphism. Genetics.

[pbio-0030193-b63] Geyer CJ, Keramidas EM (1991). Markov chain Monte Carlo maximum likelihood. Computing science and statistics. Proceedings of the 23rd Symposium on the Interface; April 21–24, 1991.

[pbio-0030193-b64] Robert CP, Casella G (2004). Monte Carlo statistical methods.

[pbio-0030193-b65] Glazko GV, Nei M (2003). Estimation of divergence times for major lineages of primate species. Mol Biol Evol.

[pbio-0030193-b66] Vignaud P, Duringer P, Mackaye HT, Likius A, Blondel C (2002). Geology and palaeontology of the Upper Miocene Toros-Menalla hominid locality, Chad. Nature.

[pbio-0030193-b67] Brunet M, Guy F, Pilbeam D, Mackaye HT, Likius A (2002). A new hominid from the Upper Miocene of Chad, Central Africa. Nature.

[pbio-0030193-b68] Wildman DE, Uddin M, Liu G, Grossman LI, Goodman M (2003). Implications of natural selection in shaping 99.4% nonsynonymous DNA identity between humans and chimpanzees: Enlarging genus *Homo*. Proc Natl Acad Sci U S A.

[pbio-0030193-b69] Chen F-C, Li W-H (2001). Genomic divergences between humans and other hominoids and the effective population size of the common ancestor of humans and chimpanzees. Am J Hum Genet.

[pbio-0030193-b70] Wright S (1951). The genetical structure of populations. Ann Eugenics.

[pbio-0030193-b71] Hudson RR, Slatkin M, Maddison WP (1992). Estimation of levels of gene flow from DNA sequence data. Genetics.

[pbio-0030193-b72] Harding RM, Fullerton SM, Griffiths RC, Bond J, Cox MJ (1997). Archaic African and Asian lineages in the genetic ancestry of modern humans. Am J Hum Genet.

[pbio-0030193-b73] Chakravarti A, Buetow KH, Antonarakis SE, Waber PG, Boehm CD (1984). Nonuniform recombination within the human β-globin gene cluster. Am J Hum Genet.

[pbio-0030193-b74] Savatier P, Trabuchet G, Faure C, Chebloune Y, Gouy M (1985). Evolution of the primate beta-globin gene region. High rate of variation in CpG dinucleotides and in short repeated sequences between man and chimpanzee. J Mol Biol.

[pbio-0030193-b75] Mishmar D, Ruiz-Pesini E, Golik P, Macaulay V, Clark AG (2003). Natural selection shaped regional mtDNA variation in humans. Proc Natl Acad Sci U S A.

[pbio-0030193-b76] Ingman M, Kaessmann H, Paabo S, Gyllensten U (2000). Mitochondrial genome variation and the origin of modern humans. Nature.

[pbio-0030193-b77] Shen P, Wang F, Underhill PA, Franco C, Yang WH (2000). Population genetic implications from sequence variation in four Y chromosome genes. Proc Natl Acad Sci U S A.

[pbio-0030193-b78] Kaessmann H, Heissig F, von Haeseler A, Paabo S (1999). DNA sequence variation in a non-coding region of low recombination on the human X chromosome. Nat Genet.

[pbio-0030193-b79] Kaessmann H, Wiebe V, Paabo S (1999). Extensive nuclear DNA sequence diversity among chimpanzees. Science.

[pbio-0030193-b80] Jaruzelska J, Zietkiewicz E, Batzer M, Cole DE, Moisan JP (1999). Spatial and temporal distribution of the neutral polymorphisms in the last ZFX intron. Analysis of the haplotype structure and genealogy. Genetics.

[pbio-0030193-b81] Jaruzelska J, Zietkiewicz E, Labuda D (1999). Is selection responsible for the low level of variation in the last intron of the ZFY locus?. Mol Biol Evol.

[pbio-0030193-b82] Thorstenson YR, Shen P, Tusher VG, Wayne TL, Davis RW (2001). Global analysis of ATM polymorphism reveals significant functional constraint. Am J Hum Genet.

[pbio-0030193-b83] Hammer MF, Garrigan D, Wood E, Wilder JA, Mobasher Z (2004). Heterogeneous patterns of variation among multiple human X-linked loci: The possible role of diversity-reducing selection in non-Africans. Genetics.

