# Supplementary material for: On the Number of New World Founders: A Population Genetic Portrait of the Peopling of the Americas
Source: PLoS Biol. 2005 May 24;3(6):e193. doi: 10.1371/journal.pbio.0030193 (PMC1131883; doi:10.1371/journal.pbio.0030193)
Supplement: Supplementary file 1 [file pbio.0030193.sd002.doc]

Supplementary information	J. Hey

**Additional Tests by Simulation - pages 1 - 6**

**Sample locations (as described in original references ) - pages 7-10**

Additional Tests by Simulation

The computer program implements a method for assessing posterior distributions for a highly parameterized model. Lengthy run times are required in part because of the need to integrate over complex nuisance parameters (i.e. one genealogy for every locus), over the course of the Markov chain simulation. While in principal the method could be used to estimate the joint maximum likelihood estimate (e.g. for the seven parameters in figure 1B) and the overall likelihood of the data given the model, the large number of parameters and the long run times mean that as a practical matter results are generally limited to marginal posterior distributions.

Testing the method requires that data sets be simulated under the model using known parameter values. This is laborious because there is not a direct relationship between the parameters used to simulate a data set and the posterior densities that are estimated from that simulated data. Rather, multiple simulated data sets generated for a common set of parameter values need to be analyzed so that a set of posterior densities can be assessed in relation to the true parameter values used for the simulations.

Some simulation results are shown in the paper. Here are provided results from a larger set of simulations used to assess the tradeoff between migration rate, and the splitting parameter, s. The simulated data sets were off a size comparable to that in the paper, and the model that was assumed is a fairly demanding one, with recent splitting time and having both population growth and population shrinkage. The parameter values were: θ_1_,= 20; θ_2_, = 5; θ_A_ = 10; *t* = 0.5; and *s* = 0.2. In other words, population 1 grew from having a population parameter of size 0.2 ×10 = 2, at t=0.5, to one with a parameter value of 20. Population 2 shrunk from a value of (1-0.2) ×10 = 8 to a value of 5.

Migration can have a large effect on the pattern of variation. In the limit of very high migration, two populations will appear to be one and samples are not expected to reveal a history of population splitting. To assess the affect of increasing amounts of migration on the estimation of other parameters, four migration rates were considered: *m_1_*=*m_2_*=0; *m_1_*=*m_2_*=0.1; *m_1_*=*m_2_*=0.5; *m_1_*=*m_2_*=1.0. Because of the changing population sizes, the effective rate of migration is changing in the time since *t*. However to get a rough sense of what these values mean in terms of the population migration rates, 2Nm (i.e. 2 × Effective population size × migration rate per gene copy per generation), we can calculate these values with respect to the ancestral population size. In this case the four rates correspond to 2N_A_m values of 0, 0.5, 2.5 and 5. These latter values are sufficiently high that they would be expected to rapidly remove most signs of population splitting. However because the splitting time is recent, it may still be possible to estimate splitting-related parameters.

In each simulation there were 10 loci, each under the infinite sites mutation model, and each with 32 samples per population. Markov chains were run with a burn-in of 100,000 steps and between 3,000,000 and 10,000,000 steps. In order to show the results of all four sets of simulations in individual graphs, the posterior distributions for each set of 10 data sets were summed.

Figure *a* shows the summed distributions for θ_1_ . In this case the curves strongly suggest that this parameter (for a population with strong growth since being recently founded) is increasingly difficult to estimate with more migration, and that the true value is consistently underestimated when migration rates are high. In contrast in figure *b* , the curves for θ_2_*,* suggest that for a population that has become smaller since founding, that migration has very little effect on the quality of the estimates.

Figures *c* and *d* show θ_A_ and *t,* respectively. For θ_A_ , the only effect of added migration appears to be a modest increase in the width of the posterior distributions. This is perhaps not surprising as in these simulations with low *t* values most of the variation in the samples arose in that ancestral population. The main shift in the curves for *t* is that more area occurs in the tail to the right, with increasing amounts of migration.

For *s* (figure *e* )a prior upper bound of 0.5 was assumed. Other simulations had shown that, particularly with migration, the posterior estimate for *s* is often quite broad and sometimes has two peaks, one below and one above 0.5. The curves suggest that *s* can be roughly estimated when migration is low, but not when migration is high.

Both migration parameters (figures *f* and *g*) generated broad curves, suggesting fairly little resolution. For the lowest migration rates the curves tend to peak at 0. For higher migration rates there were peaks in the summed distributions suggesting that the estimates, while of little confidence, are not strongly biased.

Figure Legend

Each curve in each part of the figure shows the sum of 10 posterior distributions generated from simulated data sets as described in the text above the figures. Also shown with a gray vertical bar, in figures *a,b,c,d,*and *e* are the true values of the parameters that were used in the simulations. In figures *f* and *g* the true values with colored vertical bars for each of the migration rates. The data sets for the case if *m_1_= m_2_=*0 were analyzed under a model that assumed zero migration, and so no migration curves are available in this case.

Sample descriptions for nine loci - see Table 2 of paper and original references for additional explanation

The APXL, TNFSF5 and RRM2P4 loci all used the following samples (Hammer et al. 2004)

Asian Samples - source and id #

China S. Han 66

China S. Han 67

China S. Han 68

Siberia Yakut 49

Siberia Yakut 51

New World - source and ID #

Americas U.S.A. Poarch Creek 27

U.S.A. Tohono O'Odham 25

U.S.A. Navajo 23

U.S.A. Amerindian 2

U.S.A. Amerindian 4

Mexico Mayan 17

Brazil Karatiana 12

Brazil Karatiana 13

Brazil Surui 16

Brazil Surui 14

ß-globin	(Harding et al. 1997)

Asian source and # of samples

Mongolia	24

New World source and # of samples

Nuu-Chah-Nuth from the U.S. Pacific Northwest 48

mtDNA (Ingman et al. 2000; Mishmar et al. 2003)

Asian samples, with Accession #’s

AF346971	Chukchi

AF346993	Korean

AF346991	Khirgiz

AF346973	China

AF346972	China

AF346970	Buriat

AF346979	Evenki

AY195760	Korea

New World Samples with Accession #’s

AF346984	South American Indian (Guarani)

AF347013	South American Indian (Warao)

AF347012	South American Indian (Warao)

AF347001	North American Indian (Piman)

AY195749	Native American

AY195786	Native American (Mixteca Baja)

AY195787	Native American (Navajo)

Non-recombining Y (NRY) (Hammer et al. 2003)

Asia - source and sample ID #

China Chinese	JW84

China Han	YCC66

China Han	YCC68

China Han	YCC67

Siberia Buryat	Bur76

Siberia F.Nentsi	FN11

Siberia Khant	Kha42

Siberia Selkup	Sel5

Siberia Yakut	YCC47

Siberia Yakut	YCC48

Siberia Yakut	YCC51

Siberia Yakut	YCC49

Siberia Yakut	YCC50

New World Source and Simple ID #

Arizona Navajo	YCC23

Arizona Tohono	YCC25

Brazil Karatiana	YCC13

Brazil Surui	YCC16

Brazil Karatiana	YCC12

Brazil Surui	YCC14

Brazil Surui	YCC15

USA Porch Crk	YCC27

USA Amerind	YCC2

USA Amerind	YCC3

USA Amerind	YCC4

Yucatan Mayan	YCC17

Yucatan Mayan	YCC18

Xq13.3 (Kaessmann et al. 1999)

Asia ID# and source

15-Kyrgyz

38-Buriat

43-Korean

46-Evenk

3-Chukchi

39-Chinese

40-Chinese

41-Chinese

42-Chinese

New World ID# and source

37-Warao

50-Native

56-Warao

ZFX (Jaruzelska et al. 1999)

Asia - source and # of samples

Siberian Nentsi (25)

Chinese from mainland China (25)

New World - source and # of samples

Amerindians by Ojibwa (19)

Maya (23)

Karitiana from	Brazil (16)

ATM	(Thorstenson et al. 2001)

Asia - # of samples and source

16 Han Chinese

2 Korean

2 Yakut

New World - # of samples and source

2 Karitiana

2 Surui

4 Mayans

4 Colombian Indians

2 Quechua

2 Muskogee

2 Pima

2 Navaho

References

Hammer MF, Blackmer F, Garrigan D, Nachman MW, Wilder JA (2003) Human population structure and its effects on sampling y chromosome sequence variation. Genetics 164(4): 1495-1509.

Hammer MF, Garrigan D, Wood E, Wilder JA, Mobasher Z, et al. (2004) Heterogeneous patterns of variation among multiple human X-linked loci: the possible role of diversity reducing selection in non-Africans. Genetics in press.

Harding RM, Fullerton SM, Griffiths RC, Bond J, Cox MJ, et al. (1997) Archaic African and Asian lineages in the genetic ancestry of modern humans. American Journal of Human Genetics 60(4): 772-789.

Ingman M, Kaessmann H, Paabo S, Gyllensten U (2000) Mitochondrial genome variation and the origin of modern humans. Nature 408(6813): 708-713.

Jaruzelska J, Zietkiewicz E, Batzer M, Cole DE, Moisan JP, et al. (1999) Spatial and temporal distribution of the neutral polymorphisms in the last ZFX intron. Analysis Of the haplotype structure and genealogy. Genetics 152(3): 1091-1101.

Kaessmann H, Heissig F, von Haeseler A, Paabo S (1999) DNA sequence variation in a non-coding region of low recombination on the human X chromosome. Nat Genet 22(1): 78-81.

Mishmar D, Ruiz-Pesini E, Golik P, Macaulay V, Clark AG, et al. (2003) Natural selection shaped regional mtDNA variation in humans. Proc Natl Acad Sci U S A 100(1): 171-176.

Thorstenson YR, Shen P, Tusher VG, Wayne TL, Davis RW, et al. (2001) Global analysis of ATM polymorphism reveals significant functional constraint. Am J Hum Genet 69(2): 396-412.
